# Neuropoly: An Educational Board Game to Facilitate Neurology Learning

**DOI:** 10.3389/fnsys.2021.688210

**Published:** 2021-10-06

**Authors:** Anton Raskurazhev, Polina Kuznetsova, Anastasia Evgenievna Khizhnikova, Anton Klochkov, Ilya Bakulin, Vladislav Annushkin, Marine Tanashyan, Natalya Suponeva, Elena Gnedovskaya

**Affiliations:** Research Center of Neurology (Russia), Moscow, Russia

**Keywords:** neurology education, educational games, board games, neurophobia, teaching

## Abstract

**Introduction**: Neurology is arguably one of the most difficult subjects to teach and study in the medical curriculum. Educational games (EG) may be a valid option to enhance motivation in neurology residents.

**Methods**: We developed an educational board game (Neuropoly) to assist in teaching neurology. We present here an overview of the game, as well as the results of a pilot study aimed at determining: (a) the efficacy of the game in teaching certain neurological concepts; and (b) student compliance and satisfaction with the EG.

**Results**: The pre- and post-play questionnaire scores differed significantly (3.2 ± 1.7 vs. 7.8 ± 1.6, *p* < 0.001). Our group of residents, showing an overwhelmingly positive response, very well received the game. The questions were rated as above average regarding difficulty.

**Conclusion**: The “Neuropoly” educational board game has been shown to be interesting, efficient, and motivational among first- and second-year neurology residents. Novel educational methods for complex medical disciplines should be developed, with board games being a viable and inexpensive approach.

## Introduction

Neurology may seem to the uninitiated an overly complex clinical discipline, leading to frustration and “neurophobia” among medical students and residents. Neurophobia has been described “as a fear of the neural sciences and clinical neurology that is due to the students’ inability to apply their knowledge of basic sciences to clinical situations” (Jozefowicz, 1994). The daunting task of memorizing even basic concepts is not made clearer by the sheer amount of text in most handbooks. Yet the powers of relevant clinical and fundamental neurological knowledge in successfully establishing a diagnosis may seem “magical” to adepts of other medical professions.

Education in the art of neurology is of major importance in modern healthcare given the epidemiological rise of nervous disorders and an even sharper rise in the interest in neuroscience. Neurological disorders account for 16.5% of global deaths, second only to heart disease, and are the leading cause of disability (GBD 2016 Neurology Collaborators, 2019). All this leads to an increased demand for highly qualified neurology specialists-with an emphasis on medical education. It has been postulated that teaching with appropriate didactic methodology and feedback, and plenty of practical training can improve effective learning in neurology (Ansakorpi et al., 2017). In a systematic review by McColgan et al. (2013), the importance of introducing more effective educational interventions into the teaching of neurology is underlined.

Educational games (EG) are “instructional method[s] requiring the learner to participate in a competitive activity with preset rules” (Fitzgerald, 1997). Several studies have shown the efficacy of EG in medical education (Schuh et al., 2008; Cutumisu et al., 2019).

We developed an EG (“Neuropoly”) to enhance motivation in learning among first- and second-year neurology residents at the Research Center of Neurology. We present here an overview of the game, as well as the results of a pilot study aimed at determining: (a) the efficacy of the game in teaching certain neurological concepts; and (b) student compliance and satisfaction with the EG.

There are some limits in our research work, such as the absence of medical students at our base.

## Materials and Methods

The study was performed at the Research Center of Neurology (Moscow, Russian Federation), among first- and second-year neurology residents (*n* = 51, median age 24 years [24;24], female 80.4%). Due to the fact that all residents communicated frequently with each other (including out of hours) and could unintentionally describe the game/questions/answers, we decided to opt for a “no control group” setting. No reward for playing the game was intended.

Statistical analysis was performed using Statistica software (ver. 12.0) and included descriptive statistics (mean, standard deviation, frequency), non-parametric statistics (median, interquartile range, comparison of two independent samples using Mann-Whitney test, comparison of two dependent samples using Wilcoxon signed-rank test).

The study was approved by the institutional review board, all subjects signed the informed consent form. All participants were informed of the right to refuse to participate in the study at any stage (including participation in the survey).

## Neuropoly Board Game

**Figure 1:**
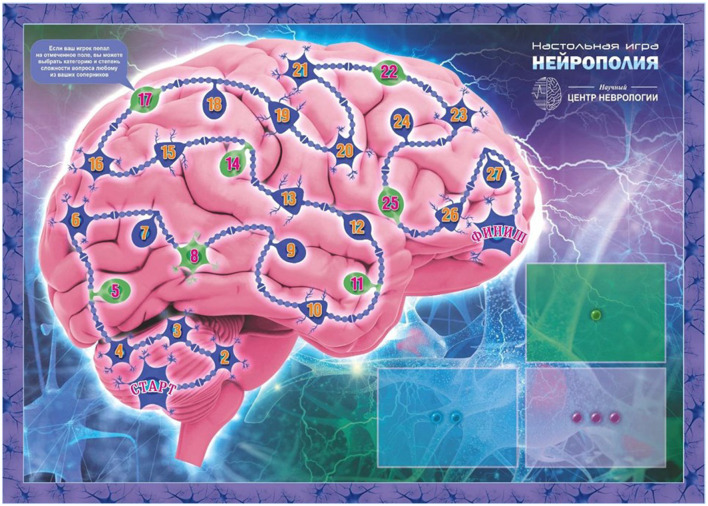
“Neuropoly” game board.

“Neuropoly” is a competitive board game, which consists of the following elements:

(a) A board (Figure 1) with spaces for players’ characters placement

(b) Three decks of question cards (30 each) marked with one (green), two (blue) or three (purple) dots corresponding to the three levels of difficulty (basic, intermediate, advanced) The distribution of questions into three groups was done with the help of three experts (all teachers of higher school) in the field of neurology, their task was to assess the severity of the question, if the opinion of two out of three professors coincided (in which group the questions should be assigned) then the question was sent to the appropriate card (basic, intermediate, advanced)

(c) Two dice (six-sided and four-sided) which determine the difficulty and the number of the question from the card, respectively

(d) Players’ characters

(e) Manual

“Neuropoly” is essentially a “racing” game for two to four players involving players asking each other questions about different topics in neurology and then checking the answers, which are written upside-down on the same cards and scoring points ("steps") for successful answers.

All cards share their structure and contain four questions corresponding to four fields of neurology: neuroanatomy, topical diagnostics, diagnosis and treatment, clinical presentation.

A list of example questions and answers is given in Table 1. The total number of questions is 360.

**Table 1:** Examples of questions corresponding to different difficulty levels in the “Neuropoly” EG.

Difficulty	Question	Answer
“Basic”	The floor of which ventricle is constituted by the rhomboid fossa?	Fourth ventricle
“Basic”	The damage to which cranial nerve results in the following: upper throat anesthesia, decrease in salivation, and loss of taste sensation to the posterior one-third of the tongue?	Glossopharyngeal nerve
“Basic”	Which antiviral drug is used in Parkinson’s disease treatment?	Amantadine
“Basic”	A common complication of subarachnoid hemorrhage, occurring on day 3–5 is‥.	Vasospasm
“Intermediate”	Which type of glial cells produces myelin in the central nervous system?	Oligodendrocytes
“Intermediate”	The Hakim-Adams triad is specific to ‥.	Normal pressure hydrocephalus
“Intermediate”	Which treatment option is used in Guillaine-Barre syndrome when plasmapheresis is contraindicated?	Intravenous immunoglobulin
“Intermediate”	How is the isolated paresis of the internal ocular muscles (pupillary sphincter and ciliary muscle) called?	Internal ophthalmoplegia
“Advanced”	Which fibers cross in Forel’s decussation?	Rubrospinal and rubroreticular tracts
“Advanced”	Parinaud’s syndrome involves damage to which structure?	Superior colliculus of the mesencephalic tectum
“Advanced”	Name a drug used for treating multiple sclerosis which modulates sphingosine-1-phosphate receptors	Fingolimode
“Advanced”	Naffziger syndrome—is an eponymic for ‥.	Scalenus anterior syndrome

The game proceeds in turns, starting with the first player. At the start of each turn the active player throws two dice, which determine: a color [six-sided dice, corresponds to the color (i.e., difficulty) of the card] and a number (four-sided dice, corresponds to the question number on the card face). The player sitting to the left then takes the top card from the chosen pile and reads out loud the corresponding question. If the active player answers correctly, he moves his player figure one or three steps on the game track (depending on the number of dots on the back of the card). If the answer is incorrect, nothing happens. If the player figure is on the green-colored spot, the active player may then choose an opponent and both the difficulty and number of the question for him. The turn then goes to the next player in clockwise order. The winner is the first player who reaches spot “28” (“Finish”). The duration of each game session depends on the number of players, with a typical game lasting between 40 and 60 min.

## Results

The study included 51 1- and 2-year neurology residents (median age 24 years [24;24], female 80.4%) who played the game for the first time in groups of four. Prior to the game session, each of the residents was asked to complete the Pre-play Questionnaire (Table 2), which included 10 questions from the game. All residents had a total of two to three game sessions in the course of 2 weeks. During this time there were no changes to their neurology curriculum and no additional reading was encouraged. After 2 weeks from the date of the first game session, each participant completed a second Questionnaire. The results were measured as “1”—for the right answer, “0”—wrong answer.

**Table 2:** Pre-play questionnaire.

	Question	Answer*
1.	Which condition is caused by oxalyldiaminopropionic acid (contained in certain plants in the legume family Fabaceae) toxicity?	Lathyrism
2.	Which drug used in multiple sclerosis treatment is a humanized anti-CD20 monoclonal antibody?	Ocrelizumab
3.	Which virus causes progressive multifocal leukoencephalopathy?	JC virus
4.	Treatment of choice for paroxysmal hemicrania is‥.	Indomethacin
5.	Repeated lobar hemorrhages with acute focal neurological signs and gradual onset of dementia are definitive of‥.	Cerebral amyloid angiopathy
6.	The pectineus muscle is innervated by ‥.	Femoral nerve
7.	Neurotransmitter released by Renshaw cells?	Glycine
8.	Transient limb weakness after an epileptic seizure is termed ‥.	Todd’s paralysis
9.	Which ipsilateral cranial nerve is involved in Weber’s syndrome?	Oculomotor nerve
10.	The alien hand syndrome phenomenon most frequently occurs in this neurodegenerative disorder - ‥.	Corticobasal degeneration

*Not included in the original Questionnaire.

There was a statistically significant difference between pre- and post-play Questionnaire scores (Table 3).

**Table 3:** Pre- and post-play questionnaire scores.

	Questionnaire 1	Questionnaire 2	*p*
Mean ± std.dev.	3.2 ± 1.7	7.8 ± 1.6	<0.001

The mean difference between Questionnaire 1 and 2 scores was 4.6 ± 1.7.

Differences between 1- and 2-year residents were observed only regarding pre-play Questionnaire 1—with second-year residents getting statistically significant higher results (4.2 ± 1.9 vs. 2.7 ± 1.3, *p* = 0.001). No gender-related differences in results were observed.

After the game, all residents were asked to complete an evaluation form (Table 4).

**Table 4:** Residents opinions about the “Neuropoly” EG.

	Question	Results
1.	Did “Neuropoly” help you memorize certain aspects of neurology?	“Yes”—96%
2.	Did you like the game? Rate 1—10 (1—did not like at all, 10—excellent)	9 [8;10]*
3.	Would you play it again? (1—yes, 0—no)	“Yes”—88.2%
4.	How difficult did you find the questions? Rate 1—10 (1—very easy, 10—very hard)	7 [6;8]*
5.	Were the rules clear enough?	“Yes”—98%
6.	Would you advise this game to your friends/colleagues? (1—yes, 0—no)	“Yes”—96%

*Data presented as median [25th percentile, 75th percentile].

Overall, the game was very well received by our group of residents, showing an overwhelmingly positive response (Table 4). The questions were rated as above average regarding difficulty, yet the rules were easy enough, offering a high replayability value.

## Discussion

Board games have been shown to positively mediate cognition (Raskurazhev et al., 2020), and as a growing modern trend may be implemented in various fields of medical science. Educational board games are a valid option for the enhancement of medical education through an increase in involvement and motivation among neurology residents.

Our results demonstrate that the “Neuropoly” EG was found by our residents to be helpful in memorizing certain aspects of neurology. This is in line with other studies on board game education in neurology, particularly in (Chaves et al., 2020), where the “Synaptic” board game was also favorably rated by medical residents. Short-term efficacy was also demonstrated in our study with a near two-fold increase between pre- and post-play Questionnaires. Overall, residents gave an excellent rating to our game—with 96% recommending it to their friends/colleagues.

The “Neuropoly” educational board game has been shown to be interesting, efficient, and motivational among first- and second-year neurology residents. Novel educational methods for complex medical disciplines should be developed, with board games being a viable and inexpensive approach.

## Data Availability Statement

The raw data supporting the conclusions of this article will be made available by the authors, without undue reservation.

## Author Contributions

AR, PK, and MT contributed to the conception and design of the study. VA organized the database. AK and AR performed the statistical analysis. AR wrote the first draft of the manuscript. AEK, IB, EG, and NS wrote sections of the manuscript. All authors contributed to the article and approved the submitted version.

## Conflict of Interest

The authors declare that the research was conducted in the absence of any commercial or financial relationships that could be construed as a potential conflict of interest.

## Publisher’s Note

All claims expressed in this article are solely those of the authors and do not necessarily represent those of their affiliated organizations, or those of the publisher, the editors and the reviewers. Any product that may be evaluated in this article, or claim that may be made by its manufacturer, is not guaranteed or endorsed by the publisher.
